# A Novel Strain of Porcine Adenovirus Detected in Urinary Bladder Urothelial Cell Culture

**DOI:** 10.3390/v6062505

**Published:** 2014-06-23

**Authors:** Urška Dragin Jerman, Marko Kolenc, Andrej Steyer, Peter Veranič, Mateja Poljšak Prijatelj, Mateja Erdani Kreft

**Affiliations:** 1 Institute of Cell Biology, Faculty of Medicine, University of Ljubljana, SI-1000 Ljubljana, Slovenia; E-Mail: urska.dragin.mf.uni-lj.si; 2 Institute of Microbiology and Immunology, Faculty of Medicine, University of Ljubljana, SI-1000 Ljubljana, Slovenia; E-Mails: marko.kolenc@mf.uni-lj.si (M.K.); andrej.steyer@mf.uni-lj.si (A.S.); peter.veranic@mf.uni-lj.si (P.V.); mateja.poljsak-prijatelj@mf.uni-lj.si (M.P.P.)

**Keywords:** porcine adenovirus, cell culture, urinary bladder, transmission electron microscopy, polymerase chain reaction, microbiological screening

## Abstract

Contamination of cell cultures is the most common problem encountered in cell culture laboratories. Besides the secondary cell contaminations often occurring in the cell laboratories, the contaminations originating from donor animal or human tissue are equally as common, but usually harder to recognize and as such require special attention. The present study describes the detection of porcine adenovirus (PAdV), strain PAdV-SVN1 in cultures of normal porcine urothelial (NPU) cells isolated from urinary bladders of domestic pigs. NPU cell cultures were evaluated by light microscopy (LM), polymerase chain reaction (PCR), and additionally assessed by transmission electron microscopy (TEM). Characteristic ultrastructure of virions revealed the infection with adenovirus. The adenoviral contamination was further identified by the sequence analysis, which showed the highest similarity to recently described PAdV strain PAdV-WI. Additionally, the cell ultrastructural analysis confirmed the life-cycle characteristic for adenoviruses. To closely mimic the *in vivo* situation, the majority of research on *in vitro* models uses cell cultures isolated from human or animal tissue and their subsequent passages. Since the donor tissue could be a potential source of contamination, the microbiological screening of the excised tissue and harvested cell cultures is highly recommended.

## 1. Introduction

Cell cultures have become indispensable tools for biological and medical research. Advances in cell culture and development of various *in vitro* models have found a number of applications in studying tissue development and function in health and disease. However, to provide reliable and reproducible results, cell cultures must be healthy and above all uncontaminated. Handling with cell cultures always poses the risk of contamination, either with eukaryotic cells from other cell cultures or, more frequently, with microbiological organisms including fungi and bacteria, and sometimes with persistent viral infections. Therefore, to maintain experiment integrity, any risks of contamination should be managed effectively.

Contamination with bacteria or fungi usually causes visible effects on cell cultures, viruses are on the contrary, due to their small size and lack of visual cues of their presence, difficult to detect by routine light microscopy (LM) and thus might easily be overlooked [1]. Frequently contamination with viruses remains unrecognized, unless viral infection leads to cytopathological changes of the cultured cells, such as atypical cell morphology or increased cell death. The cell culture laboratory environment, the personnel or rarely already contaminated cell lines could be the source of viruses. However, most commonly, the viral infection originates from infected donor animals, either by serum or when using an animal tissue as a source of cells for primary and subsequent cell cultures [1].

Adenoviruses (AdVs) are non-enveloped, icosahedral viruses, with a linear double stranded DNA genome that can infect all five major vertebrate classes [2]. Porcine adenoviruses (PAdVs) are classified within the genus *Mastadenovirus* in the *Adenoviridae* family [2], and are regarded as low grade pathogens, infecting the porcine populations worldwide. They often do not cause any disease [3], or the infection is only manifested in a milder diarrhea [4] or respiratory signs [5], with no other associated clinical symptoms. There are at least five types of PAdV circulating in domestic pig populations internationally [6], among which PAdV types 1 to 3 are closely related, whereas types 4 and 5 are less similar, both to this group and to each other [2].

AdVs enter the host cell by receptor-mediated endocytosis. They can bind to one of the adenovirus receptors, e.g., coxsackievirus and adenovirus receptor (CAR) at the cell surface and locally activate the αv superficial cell integrins, which triggers the clathrin-mediated endocytosis [7,8]. Once inside the endosome, they rapidly lyse the endosomal membrane and escape to the cytosol. By trafficking along the microtubules they reach the nucleus, where they bind to the nuclear envelope and release the viral genome into the nucleus through the nuclear pore [9]. After the selective transcription and translation of viral genes, the AdVs assembly in the nucleus and leave the host cell via the induced cell lysis [10].

The current study describes the detection of AdV in subsequent cultures of normal porcine urothelial (NPU) cells isolated form urinary bladders of domestic pigs (*Sus scrofa domestica*). To detect the AdV, first the negative staining of NPU cell cultures was performed. The PAdV was further confirmed by polymerase chain reaction (PCR) amplification of AdV hexon gene and additionally characterized by transmission electron microscopy (TEM). According to the cell culture origin and the unique molecular characteristics of the analyzed AdV isolate, it was designated as PAdV-SVN1 (Porcine Adenovirus strain SVN1). Since most PAdV infections are asymptomatic, the PAdV detection methods described herein are advisable always when establishing the primary and subsequent cell cultures from porcine tissue. Overall, only early recognition of viral contamination can reduce the risk of transferring the infection also to the other cell cultures in the cell laboratory.

## 2. Materials and Methods

### 2.1. Porcine Urinary Bladders and Establishment of Primary and Secondary NPU Cell Culture

The experiments were approved by the Veterinary Administration of the Slovenian Ministry of Agriculture and Forestry in compliance with the Animal Health Protection Act and the Instructions for Granting Permits for Animal Experimentation for Scientific Purposes.

Porcine urinary bladders (*n* = 7) were obtained from a local slaughterhouse. The urine, urothelial, connective, and muscle tissue were tested for presence of adenoviruses with PCR. For harvesting of primary and subsequent NPU cell cultures, porcine urinary bladder was cut in large segments and NPU cells were gently scraped from urothelium, filtered through the 40 µL Cell Strainer (BD Falcon, Heidelberg, Germany), collected and seeded onto polystyrene Tissue Culture Flasks (TPP, Trasadingen, Switzerland) at a density of 2 × 10^5^ viable cells/cm^2^. At 80%–100% confluence, the NPU cells were harvested with TripLE™ Select (Gibco, Life technologies, Wien, Austria) and reseeded onto new Tissue Culture Flasks. Cells were sub-cultured until the XIII passage. The NPU cell cultures were cultured in UroM medium, which does not allow the growth of fibroblasts [11] and is adapted for the growth of porcine urothelial cells, at 37 °C in a 95% humidified atmosphere of 5% CO_2_ in air as in [12,13]. The medium was changed on alternate days. The UroM medium consisted of equal parts of MCDB153 medium (Sigma-Aldrich, Taufkirchen, Germany) and Advanced-Dulbecco’s modified essential medium (Invitrogen, Life technologies, Wien, Austria), supplemented with 2.5% fetal bovine serum (Gibco, Life technologies, Wien, Austria), 0.1 mM phosphoethanolamine (Sigma-Aldrich, Taufkirchen, Germany), 15 µg/mL adenine (Sigma-Aldrich, Taufkirchen, Germany), 0.5 µg/mL hydrocortisone (Sigma-Aldrich, Taufkirchen, Germany), 5 µg/mL insulin (Sigma-Aldrich, Taufkirchen, Germany), 4 mM glutamax (Gibco, Life technologies, Wien, Austria), 100 µg/mL streptomycin and 100 U/mL penicillin.

To confirm the presence of adenovirus, the NPU cells of I, X, XI and XIII passages were analyzed by PCR. NPU cells of the XIII passage were also prepared for negative staining and TEM of ultrathin sections.

### 2.2. Negative Staining Electron Microscopy

For negative staining NPU cell cultures were frozen, thawed three times and clarified by centrifugation at 1000× *g*. Clarified supernatant was ultracentrifuged at 100,000× *g* for 1 h. The resulting pellet was resuspended in saline solution, placed onto formvar (SPI Supplies, West Chester, PA, USA) coated with carbon stabilized grids and negatively stained using phosphotungstic acid. The grids were examined with TEM (JEM-1200 EX II, JEOL, Tokyo, Japan) at 80 kV.

### 2.3. DNA Extraction and PCR

For nucleic acid extraction, NPU cell cultures were frozen and thawed three times. Volume of 400 µL suspension was used for total viral nucleic acid extraction with iPrep™ PureLink^®^ Virus Kit (Life technologies, Invitrogen Division, Carlsbad, CA, USA). Extracted nucleic acid was stored at −80 °C.

Adenoviral nucleic acid was detected by semi-nested touchdown polymerase chain reaction (TD-PCR) via broad-spectrum primers as already described [14]. Briefly, 5’ region of AdV hexon gene was amplified using MaAdF1/R, AtAdF1/R primer pairs and MaAdF2/R, AtAdF2/R primer pairs for semi-nested TD-PCR, using Tfi polymerase enzyme system (Life technologies, Invitrogen Division). Identical TD-PCR programs were used for both rounds of semi-nested amplification as follows: 94 °C for 4 min, followed by 10 cycles of 94 °C for 30 s, 65 °C for 30 s (with a decrement of 1 °C per cycle), and 72 °C for 1 min. An additional 30 cycles were completed as follows: 94 °C for 30 s, 55 °C for 30 s, and 72 °C for 1 min, finishing with a 72 °C, 7-min extension. PCR products were confirmed based on PCR amplified fragments that can vary between 599 and 725 bp in size because of amplification of hypervariable region V1 [15]. Fragments were visualized in the agarose gel electrophoresis (1.5%) and SYBR Safe staining (Life Science, Invitrogen Division, Carlsbad, CA, USA).

For accurate characterization and classification of our isolate, a DNA-dependent DNA polymerase gene (polymerase gene) region was included in molecular analysis. The partial polymerase gene was amplified with consensus primer pair polFouter/polRouter [16]. A fragment of 553 nucleotide sequence was amplified following the protocol published by Wellehan *et al.* [16]. As positive control we used human adenovirus (HAdV) DNA isolated from a clinical sample. As negative control, the VII passage of our previously prepared NPU cells originating from another non-infected urinary bladder was analyzed. For the inhibition control, an eukaryotic 18S assay was carried out (Life Technologies, Applied Biosystems Division, Foster City, CA, USA), using OneStep real-time RT-PCR system (Life Technologies, Invitrogen Division, Carlsbad, CA, USA).

### 2.4. Sequencing

To confirm the specificity of the PCR product, amplified MaAdV hexon gene and polymerase gene fragments were purified with Wizard SV Gel and PCR Clean-Up system (Promega, Madison, WI, USA). All genome fragments were directly sequenced with initial PCR primers, used in the first round TD-PCR of the MaAdV hexon gene and PCR of polymerase gene amplification reaction. For the sequencing reaction ABI PRISM BigDyeTM 3.1 terminator Cycle Sequencing Ready Reaction Kit (Applied Biosystems) was used in a 20 µL reaction and purified with BigDye Xterminator Purification Kit (Applied Biosystems). Sequence data were generated in ABI 3500 Genetic Analyser (Life Technologies, Applied Biosystems Division, Foster City, CA, USA).

Nucleotide sequences were aligned and assembled in CLC software (CLC Bio, Aarhus, Denmark) [17] and deposited in GenBank under accession number KJ499459 (hexon gene), KJ933482 (polymerase gene).

### 2.5. Phylogenetic Analysis

The hexon and polymerase gene fragments were further included in the phylogenetic analysis. Deduced amino acid sequences of both genes were prepared and additional amino acid sequences of representative strains from GenBank were selected to ensure accurate phylogenetic analysis. Multiple alignments were performed and Neighbor-Joining phylogenetic trees were constructed with bootstrap test of 1000 replicates, using Mega software package version 5.2.2. [18].

### 2.6. Ultrathin Section Electron Microscopy

NPU cells of the XIII passage were seeded onto porous membranes (BD Falcon) at a density of 2 × 10^5^ viable cells/cm^2^. After three weeks in culture, NPU cells were fixed with 4% (w/v) paraformaldehyde and 2.5% (v/v) glutaraldehyde in 0.1 M cacodylate buffer, pH 7.4 for 2 h 45 min. The fixation was followed by overnight rising in 0.33% sucrose in cacodylate buffer and a post-fixation in 1% (w/v) osmium tetroxide for 1 h at 4 °C. The samples were afterwards dehydrated in a graded series of ethanol and embedded in Epon (Serva Electrophoresis, Heidelberg, Germany). Ultrathin sections were contrasted with uranyl acetate and lead citrate and examined with TEM (Philips CM100, Eindhoven, Netherlands) at 80 kV.

## 3. Results

### 3.1. Morphological Changes of Porcine Urothelial Cell Culture

**Figure 1:**
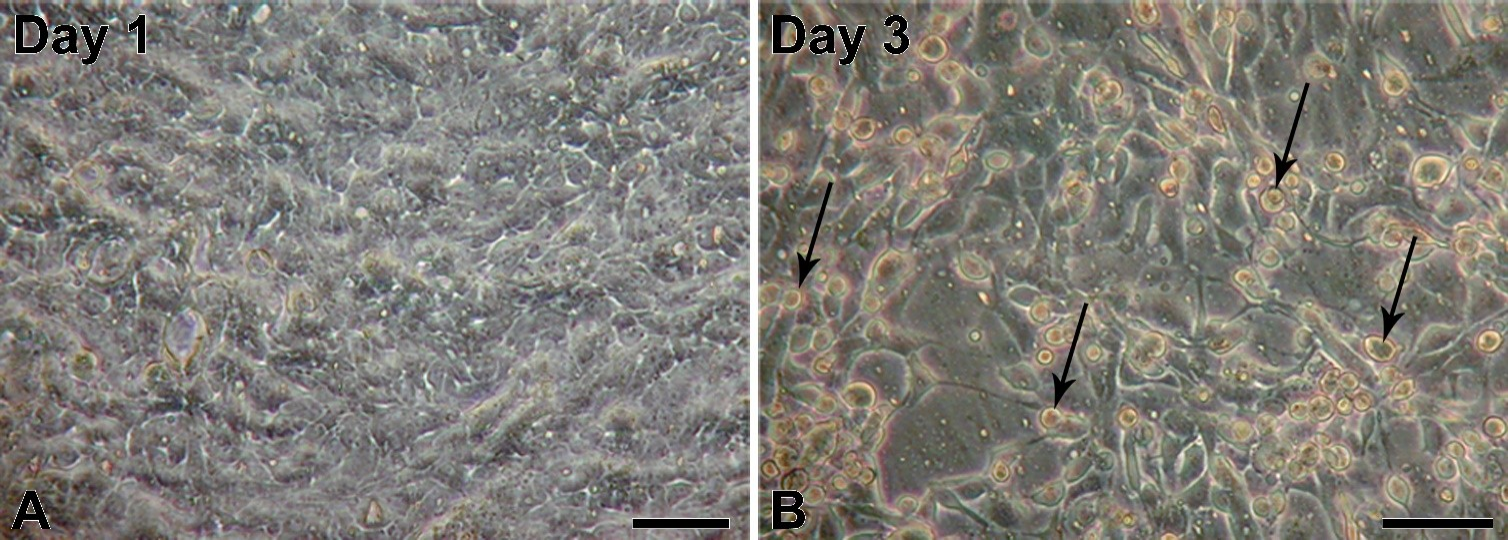
Micrographs of normal porcine urothelial (NPU) cell cultures of the XIII passage on porous membranes on a first (**A**) and third (**B**) day after seeding at a density of 2 × 10^5^ viable cells/cm^2^. Note the fast cell attachment on the first day, where NPU cells are almost confluent, and an increased cell desquamation on a third day after seeding, where numerous rounded and detached NPU cells can be seen (arrows). Scale bars: 100 µm.

Primary and the first passages (till V passage) of secondary NPU cells seeded onto culture tissue flasks had epithelial-like morphology, as expected (Figure 1A). The cells attached and proliferated normally and no unusual desquamation was noted. Following the few initial passages, increased desquamation after the first days of seeding was observed (Figure 1B), which was even more pronounced after the weekends, when medium was not changed for a longer period of time. 

Afterwards, the cell proliferation was slower and cells had difficulties in reaching the confluence. The morphology of NPU cells changed, as they become more fibroblast-like, no longer forming islands characteristics for the epithelial cell cultures. Additionally, a greater amount of cell debris was noted in the medium.

### 3.2. Negative Staining Revealed Adenovirus

**Figure 2:**
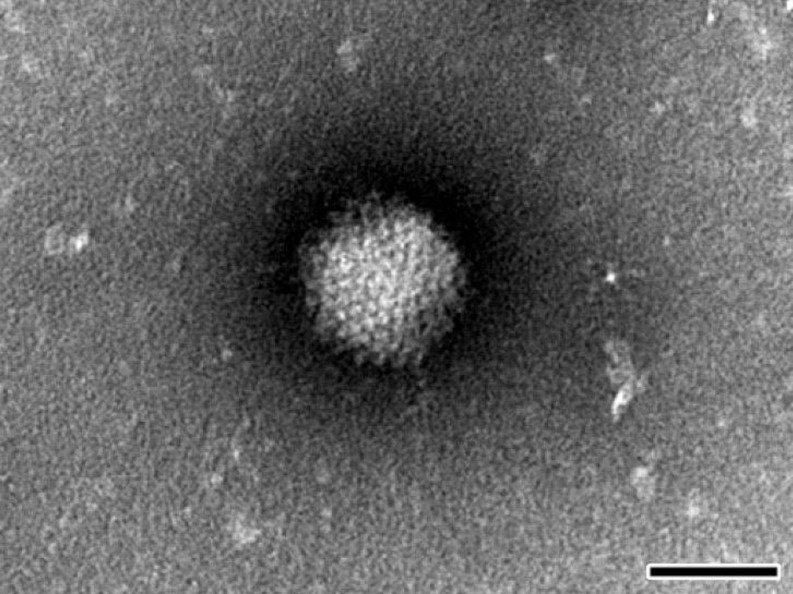
Transmission electron micrograph of negatively stained adenovirus from NPU cell culture suspension. The size and appearance of the viral particles are characteristics for the *Adenoviridae* family. Scale bar: 50 nm.

After the viral contamination was suspected, the NPU cell cultures were first prepared for negative staining electron microscopy. According to the characteristic icosahedral morphology and size of approximately 90 nm, the adenoviruses were identified (Figure 2).

### 3.3. Nucleotide Sequence Analysis Confirmed Novel Adenovirus Strain

**Figure 3:**
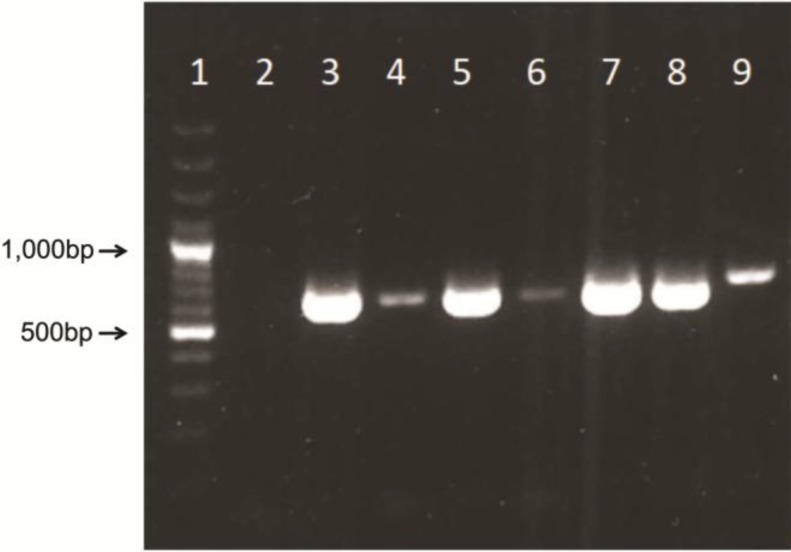
Gel electrophoresis showing 632 bp polymerase chain reaction (PCR) products of the amplified hexon gene fragment. **Lane 1**: molecular mass marker (DNA ladder 100 bp plus); **lane 2**: negative control; **lane 3**: passage XI of NPU cell culture; **lane 4**: passage XI of NPU cell culture, 10-fold dilution; **lane 5**: passage XIII of NPU cell culture; **lane 6**: passage XIII of NPU cell culture, 10-fold dilution; **lane 7**: passage X of NPU cell culture; **lane 8**: passage X of NPU cell culture, 10-fold dilution; **lane 9**: positive control (HAdV DNA).

To confirm and further determinate the adenoviral genus and to exclude the possible contamination with human adenoviruses, NPU cell cultures were prepared for PCR. The adenoviral DNA was confirmed with TD-PCR in NPU cell cultures of the passages X, XI and XIII, but not in the passage I. Likewise, adenoviral DNA was not confirmed in NPU cell cultures of the VII passage (negative control), which were primarily established from the non-infected urinary bladder (Figure 3). Also, no indication of PCR inhibition was observed as 18S assay resulted positive in all tested samples. After sequence analysis and BLAST search in GenBank, the highest similarity in nucleotide sequence (96%) was found with recently described novel porcine adenovirus strain PAdV-WI [14]. Other adenovirus genotypes shared lower identities as shown in Table 1.

**Figure 4:**
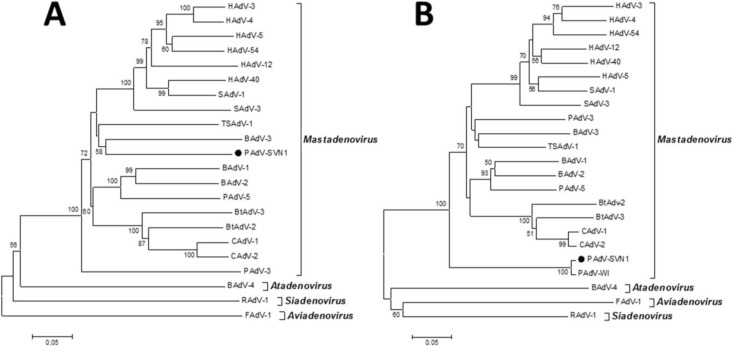
Phylogenetic tree of partial polymerase (**A**) and hexon (**B**) deduced amino acid sequences. Neighbor-Joining phylogenetic trees were constructed with bootstrap test of 1000 replicates, showing percentages next to the branches (values below 50 are not shown). The representative strains from GenBank: HAdV-3, DQ086466; HAdV-4, AY487947; HAdV-5, AC_000008; HAdV-12, X73487; HAdV-40, L19443; HAdV-54, AB333801; SAdV-1, AY771780; SAdV-3, AY598782; TSAdV-1, AC_000190; BAdV-1, NC_006324; BAdV-2, AC_000001; BAdV-3, AC_000002; BAdV-4, AF036092; PAdV-3, AF083132; PAdV-5, AF289262; PAdV-WI, JF699045; BtAdV-3, GU226970; BtAdV-2, JN252129; CAdV-1, AC_000003; CAdV-2, AC_000020; RAdV-1, EU715130; FAdV-1, AC_000014. HAdV—human adenovirus, SAdV—simian adenovirus, TSAdV—tree shrew adenovirus, BAdV—bovine adenovirus, PAdV—porcine adenovirus, BtAdV—bat adenovirus, CAdV—canine adenovirus, RAdV—raptor adenovirus, FAdV—fowl adenovirus.

The phylogenetic analysis of the hexon and polymerase gene regions indicated that the isolate PAdV-SVN1, as we named our isolate, was only distantly related to other representatives of the *Mastadenovirus* genus, currently available in GenBank (Figure 4A,B). According to the polymerase phylogenetic analysis (Figure 4A) the PAdV-SVN1 was not closely related to any of the PAdV strains available in GenBank. However, the hexon phylogenetic analysis (Figure 4B) showed that PAdV-SVN1 clustered together with the PAdV-WI, that is clearly separated from other *Mastadenovirus* representative strains, which was also strongly supported by a high bootstrap value. According to the data obtained with phylogenetic analysis it was not possible to assign the isolate PAdV-SVN1 to a specific species within the *Mastadenovirus* genus as many representatives were not available in GenBank for these two genome regions.

Additionally, the urine, urothelial, connective, and muscle tissue of porcine bladders were collected and tested for presence of adenoviruses with hexon gene specific PCR. They were all found negative (data not shown).

**Table 1:** Nucleotide (NT) and deduced amino acid (AA) sequence identities of the hexon gene fragment (599–725 bp) between Slovenian isolate porcine adenovirus (PAdV)-SVN1 and the most identical strains from GenBank.

Strain	Species within the *Mastadenovirus* Genus	GenBank Accession Number	Sequence Identity (%)	
			NT	AA
Porcine adenovirus Wisconsin isolate PAdV-WI	Unclassified *Mastadenovirus*	JF699045.1	96	95
Bat adenovirus TJM	*Bat mastadenovirus A*	GU226970.1	61	59
Tree shrew adenovirus 1	*Tree shrew mastadenovirus A*	AF258784.1	65	61
Porcine adenovirus 3	*Porcine mastadenovirus A*	U34592.1	62	56
Porcine adenovirus 5	*Porcine mastadenovirus C*	NC_002702.1	59	57

### 3.4. Intracellular Localization of Adenovirus and Ultrastructural Characteristics of Infected Urothelial Cells

**Figure 5:**
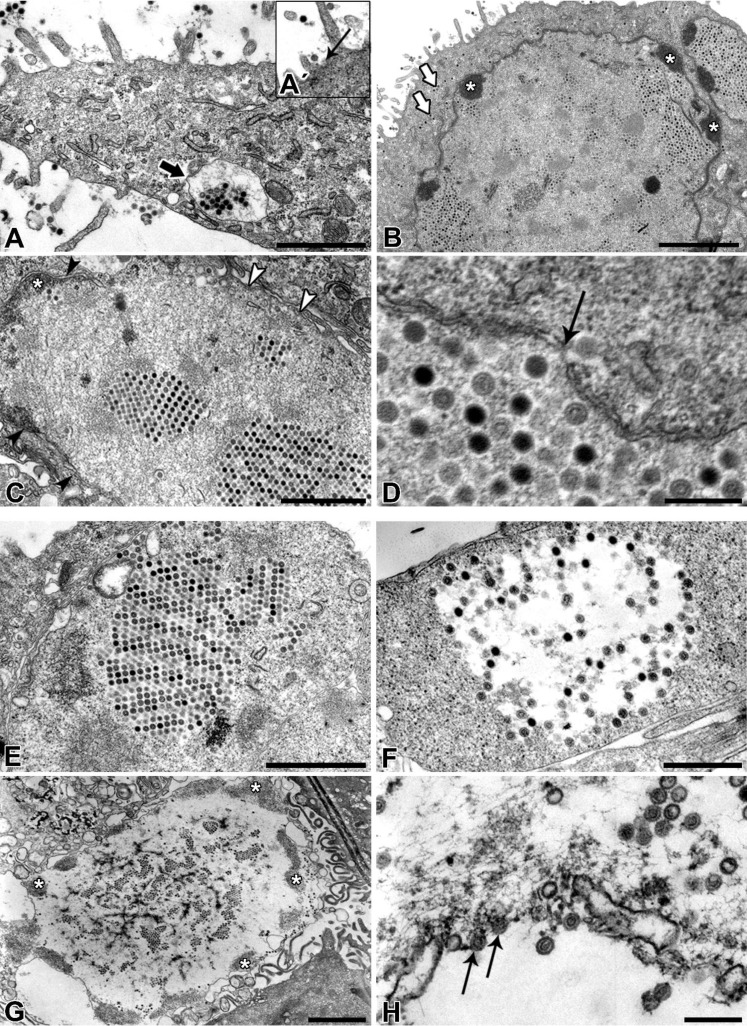
Transmission electron micrographs of PAdV-SVN1 in NPU cells isolated from porcine urinary bladder. PAdVs enter the NPU cells by endocytotic uptake (arrow on **A´**). After the endocytosis, the virions are found in late endosomes (**A**) or sporadically scattered in the cytosol (arrows on **B**). After the cell internalization, the virions are found in the nucleus, organized in larger paracrystalline arrays or spread throughout the nucleus in smaller clusters (**B**). Commonly the condensed chromatin at the nuclear envelope of infected NPU cells is noted (asterisks on **B** and **C**). The possible mechanism of nuclear escape includes the separation of nuclear envelope and merger of the nucleus full of virions with the cytosol (black arrowheads on **C** indicate the nuclear envelope, white ones the plasma membrane), or maybe even the individual release of virions through the nuclear pore (arrow on **D**). After re-entry to the cytosol, the virions are found both, in the large paracrystalline arrays (**E**) or randomly distributed throughout the cytosol (**F**), with frequently observed degradation of the cytoplasm at the site of their presence (**F**). At this point, the initiation of NPU cell necrosis can be observed. With the progress of cell lysis, the cell detaches from the epithelium, *i.e.*, urothelium (**G**); the nucleus disintegrates and condensed chromatin is moved near the plasma membrane (asterisks on **G**). At the final stage of necrosis, virions leave the cell through the lysed plasma membrane (arrows on **H**). Scale bars: (A, C and E) 1 µm; (B) 20 µm; (D and H) 200 nm; (F) 500 nm, (G) 2 µm.

Following the adenoviral molecular classification, ultrathin epon sections were prepared to analyze ultrastructural characteristics of PAdV-SVN1, its intracellular localization, and ultrastructural characteristics of the infected NPU cells. Virions were predominantly found in the nucleus, but have also been demonstrated in the cytosol and in the intercellular or extracellular space of NPU cells (Figure 5). Regarding the latter, virions were often found between the lateral plasma membranes of the adjacent NPU cells, or among the microvilli of the NPU cells neighboring the necrotic desquamated NPU cells (Figure 5A).

The viral endocytosis into NPU cells (Figure 5A´) or virions surrounded by the endosomal membrane (Figure 5A) were observed very rarely. Instead, the individual virions were often found in the cytosol of NPU cells (Figure 5B). When found in the nucleus, virions were usually arranged in the lattice-like paracrystalline arrays (Figure 5B,C), within which some of them had an electron-dense, while others an electron-lucent core (Figure 5D). Frequently, the nuclei of infected NPU cells were unusually large with characteristic pattern of condensed chromatin at the nuclear envelope (Figure 5B). In the cytosol, virions were usually arranged in the paracrystalline arrays (Figure 5E), although often noted also randomly scattered throughout the cytosol (Figure 5F). Repeatedly, the degradation of the cytoplasm at the site of the virions was found (Figure 5F). The majority of the infected NPU cells was at the initial stages of necrosis or already lysed and detached from the urothelium. From such NPU cells, the infectious virions were released into the extracellular through the damaged plasma membrane (Figure 5H).

## 4. Discussion

HAdV is most frequently associated with respiratory infections, hemorrhagic cystitis and gastroenteritis, particularly in infants and young children [19]. Similarly, PAdV in piglets often causes respiratory and gastrointestinal infections [4,5]; however, to our knowledge, infections of porcine urinary bladder have not been reported yet. The obtained porcine urinary bladders did not show any visible signs of infection; therefore, we assume that the adenoviral infection was milder and without clinical signs. First passages of NPU cells harvested from the infected urinary bladder showed normal epithelial morphology. Interestingly, also with the PCR, the adenoviral contamination of the NPU cells of the first passage was not detected, suggesting on a very low viral load just after the cell harvest. However, with cell subculturing the amount of infectious viruses seemed to increase, which resulted in NPU cell desquamation and their alteration into fibroblast-like morphology. Additionally, adenoviral DNA in the X and the following passages was successfully detected. Many factors, viral and hosts can contribute to the regulation of virus replication cycle. For the dynamic of viral load through the cell culture passages, a more sensitive method, like real-time PCR should be used, including the normalization of the cell number.

For adenoviral detection and identification different approaches can be used: the conventional methods, which include TEM, viral antigen detection and virus isolation in cell culture, and methods based on molecular techniques that focus on the amplification and detection of the viral genome [20]. In the last decades TEM has made a major contribution to virology, including the discovery of many viruses and enlightenment of the fundamental virus-host cell interactions [21]. Even though it is nowadays gradually being overruled by the more sensitive molecular detection approaches and live cell imaging fluorescence based imaging techniques [22], it still remains one of the inevitable identification tools in virology as it enables fast morphology-based identification of viruses [23] and provides the insight into the viral life cycle and its influence on a host cell fate [21]. The PCR is frequently referred to as a gold standard for molecular detection of nucleic acids in virology [24]. The method is highly sensitive and accurate, and is especially applicable when dealing with noninfectious viruses [20]. However, one of the major challenges for the development of a sensitive and universal PCR capable of detecting all viral strains still remains the high degree of heterogeneity among the various serotypes of the viral family [25]. Additionally, the molecular approach lacks the information of viral infectivity.

Considering all of the above, the most effective way to detect and fully characterize a virus is the combination of different approaches. In the present study the suspicion of viral contamination of NPU cells isolated from porcine urinary bladder, was confirmed with conventional (TEM) and molecular based (PCR) methods. Initially, TEM (negative staining) was used to detect and morphologically identify the virus belonging to the *Adenoviriade* family. Later on, the detailed classification of the adenoviral isolate was made by nucleotide sequence analysis. For this purpose a broad range of adenovirus primers was used, enabling successful amplification of the hexon and polymerase genes of various types of mammal adenoviruses. The sequencing and sequence analysis of the amplified hexon gene fragment of adenovirus isolate showed the highest similarity to recently described novel porcine adenovirus strain PAdV-WI, which was so far detected only in pen wash water of newborn to finisher pigs and has been proposed as a prototype of a new species in the *Mastadenovirus* genus [14]. According to the short nucleotide sequence of the hexon gene analyzed in this study and the comparison to the initially recognized PAdV-WI, both strains showed a clear separation from other, even the most similar representatives in the *Mastadenovirus* genus obtained from the GenBank (Figure 4B, Table 1). For the polymerase gene fragment similar pattern was observed. Strain PAdV-SVN1 was distantly related to other *Mastadenovirus* strains (Figure 4A). Unfortunately, not all species representatives within the *Mastadenovirus* genus were available for both of the analyzed genome regions. Thus, clear classification of PAdV-SVN1 as well as PAdV-WI is not possible until the whole genome sequence is available and comparable analysis with all the representative strains of *Mastadenovirus* genus is performed.

Afterwards, the TEM of ultrathin sections additionally confirmed the ultrastructure and life-cycle characteristic for AdVs. Nevertheless, according to our TEM results it remains unclear how the virions left the nucleus and re-entered the cytoplasm. We assume that the virions caused degradation of the nuclear envelope leading to the enhanced transfer of virions between nucleus and cytoplasm or they even escaped from nucleus through the nuclear pores.

Overall, an interdisciplinary approach gave us a holistic view of the viral ultrastructure, its genotype and interactions with the host cell. The present study is the first describing the isolation of the novel strain of porcine adenovirus lineage most probably originating from the urothelial tissue intended for use in the cell culture experiments. Thus when establishing cell cultures from animal or human tissue one should consider animal or human tissues as a potential source of silent viral infection, which can become apparent only after few passages in culture. The establishment and cultivation of cell cultures can be a demanding, time consuming, and costly expenditure. To avoid such cultivation of seemingly uninfected cell cultures, we propose that the ultrastructural and molecular examination of tissues and harvested cells should be an essential part in providing uncontaminated healthy cell cultures that ensure reliable and reproducible results.

In conclusion, since cell culture contamination is a frequently encountered problem, the only way to keep cell cultures as a trustworthy research tool is the effective management and prevention of its contamination. The animal tissue is a potential source of contamination, thus when choosing donor animals special care should be taken to choose only healthy animals from controlled breeding that have been regularly inspected by the veterinarian.

The PAdV-WI adenoviral strain has been just recently described, and hence many questions regarding its infectivity are still to be answered. To elucidate the risk of PAdV-WI and PAdV-SVN1 infections, screening of the pig stool and urine samples for the presence of these viruses, and also seroprevalance study on different age groups of pigs in order to determine the age group of pigs with high infection rate should be performed. Nevertheless, although the donor animal or human has no clinical signs of infection, in order to avoid potential viral contamination a multi-step approach, combining molecular and electron microscopy detection techniques is highly recommended always when considering establishing the cell culture *de novo*.
